# Biomarkers of Cognitive Training Effects in Aging

**DOI:** 10.1007/s13670-012-0014-5

**Published:** 2012-04-19

**Authors:** Sylvie Belleville, Louis Bherer

**Affiliations:** 1 Centre de Recherche, Institut Universitaire de Gériatrie de Montréal, 4565 Chemin Queen Mary, Montréal, Quebec H3W-1 W5 Canada; 2 Département de Psychologie, Université de Montréal, Montréal, Quebec Canada; 3 Département de Psychologie, Université du Québec à Montréal, Montréal, Quebec Canada

**Keywords:** Biomarkers, Cognitive training, Cognitive intervention, Brain imaging, Aging, Mild cognitive impairment, Alzheimer’s disease, fMRI, Structural imaging

## Abstract

An increasing number of studies have relied on brain imaging to assess the effects of cognitive training in healthy aging populations and in persons with early Alzheimer’s disease or mild cognitive impairment (MCI). At the structural level, cognitive training in healthy aging individuals has been associated with increased brain volume, cortical thickness, and density and coherence of white matter tracts. At the functional level, task-related brain activation (using fMRI and PET) and fluorodeoxyglucose positron emission tomography (FDG-PET) were found to be sensitive to the effects of training. In persons with MCI, cognitive training increased brain metabolism and task-related brain activation, whereas healthy older adults showed patterns of increased and decreased activation. Further studies are required to generalize these findings to larger groups and to investigate more diverse training protocols. Research will also need to address important methodological issues regarding the use of biomarkers in cognitive aging, including reliability, clinical validity, and relevance to the pathophysiological process.

## Introduction

Cognitive training is designed to restore, increase, or optimize capacities in persons suffering from cognitive impairment [1, 2••]. Many studies have used strategy memory training to enhance episodic memory in older adults. Multimodal training, which combines cognitive training with psychosocial or other activities, has also gained popularity because it is often viewed as more ecological and potentially more clinically relevant. Alternatively, a great deal of studies have used specific training protocols to assess in more detail the cognitive processes that show residual plasticity as the brain matures. Many of these have reported positive effects of cognitive training in healthy older adults [3, 4] and in persons with mild cognitive impairment (MCI) [2••, 5, 6••, 7, 8], a transitional stage between normal aging and dementia. More recently, an increasing number of studies have relied on biological measures, mostly brain imaging, to assess the effect of cognitive training in aging. An analysis of the implications and implicit assumptions underlying this approach is therefore timely, particularly with the increasing importance and appeal for the identification of biomarkers.

A biomarker is a measurement used as an indicator of normal biological or disease processes. Biomarkers should meet the criteria of being a biologically plausible measure of the disease or condition and sharing a coherent relation with the disease progression and severity. For example, brain imaging allows for the measurement of structural and functional changes that are characteristic of Alzheimer’s disease (AD) and that correlate with disease progression and severity. Brain imaging measures are also modified by the normal aging process and are related to cognitive changes that occur with age. A biomarker also can be used as “surrogate marker.” Surrogate markers are used in therapeutic trials as clinically meaningful measures of the effect of a therapy [9, 10]. Surrogate biomarkers are used when optimal clinical outcomes (for example, progression to dementia, or death) might be undesired, require too long a follow-up, or necessitate too large a sample. By using biomarkers, one can design a study where therapeutic effects are measured in a more feasible and timely manner. In the context of cognitive training, biomarkers also can provide invaluable information on the mechanisms through which an intervention enhances cognition. In addition, biomarkers can be used to quantify cognitive and neural plasticity by revealing cognitive and neural compensatory mechanisms, or by indicating patterns of changes that suggest increased efficiency of information processing in aging.

Not all biological measures are appropriate biomarkers and similarly, not all biomarkers are appropriate surrogates of training efficacy. Biomarkers need to be sensitive to the disease and to correlate with progression and severity, and their biological relation with the disease must be at least partially understood. Surrogate markers are typically biomarkers of the disease, but they also need to be sensitive to change and reliable over time, and their mechanisms should be relevant to the therapeutic effect that they are expected to indicate. In this review, we will examine structural and functional brain imaging as potential surrogate markers for cognitive training effects. We will discuss studies that have used brain imaging as a marker of cognitive training effects in healthy aging and in MCI, the early stage of AD. We also will address some challenges, limitations, and implications of using brain imaging as biomarkers of cognitive training effects in aging populations.

## Structural Imaging as a Biomarker of Cognitive Training

Structural brain imaging provides quantitative information on brain structure, including whole-brain volume, regional grey matter volumes, cortical thickness, and indicators of white matter integrity and microstructure. Brain imaging techniques have provided valuable information regarding the effects of aging on brain structure. Such studies have found that brain volume decreases with age and that those structural changes accelerate, with an annual decline of 0.35 % in older adults, compared to 0.12 % in young adults (see Dennis et al. [11] and Raz [12] for reviews). Normal aging does not alter all cognitive functions at the same time in the course of aging, and likewise, some brain regions are more sensitive to age than others. The caudate, cerebellum, hippocampi, and association cortices show the largest volume loss. In addition, aging brings changes in white matter integrity, with greater changes occurring after the seventh decade, and those are localized preferentially in the anterior regions (frontal and prefrontal).

Training-related structural changes often have been reported in younger adults and middle-aged participants, but very few studies have been published on the structural modifications produced by cognitive training in older adults. In one of these studies, Boyke et al. [13] showed with voxel-based morphometry (VBM) that grey matter volume increased in older adults after 3 months of learning classic three-ball cascade juggling. Changes were observed in the middle temporal area (V5) of the visual cortex, in the left hippocampus, and in the nucleus accumbens bilaterally. Interestingly, the training-related changes found in the hippocampus and nucleus accumbens were not observed in young adults. This study suggests that training can induce considerable structural plasticity in older adults, although older adults showed slightly smaller changes than younger adults. It is of note, however, that the authors did not find any correlation between changes in grey matter and performance or exercise intensity.

In a more recent study, Engvig and colleagues [14••] examined the effects of memory training on cortical thickness in middle-aged and elderly healthy volunteers. After 8 weeks of memory training using the method of loci, participants in the training group showed improved source memory and regional increases in cortical thickness of the lateral orbitofrontal cortex bilaterally and right fusiform cortex. Changes in cortical thickness in the right fusiform and lateral orbitofrontal cortex correlated positively with improvement in source memory, indicating that those structural changes are relevant markers of such cognitive changes. The training also had an impact on the alterations found in the white matter microstructure [15••]. Using diffusion tensor imaging (DTI), the authors found reduced fractional anisotropy (FA) in the frontal areas of untrained older adults, whereas no change was found in those who received memory training. Training-associated effects on FA seemed to be accompanied by a relative decrease in radial diffusivity, which might indicate a role for myelination in white matter plasticity. In line with other DTI studies showing that changes in myelination lead to FA changes in DTI, findings by Engvig et al. [14••, 15••] suggest that training protects against age-related reductions in myelination. In this study, memory improvements were correlated with changes in anterior FA, providing support for DTI as a valid surrogate marker for the effects of cognitive training. Lovden et al. [16] also found increased FA (and decreased mean diffusivity) in older adults after a multidimensional program with repeated practice on working memory, episodic memory, and perceptual speed tasks. These changes were found in the anterior portion of the corpus callosum but were not correlated with cognitive improvement.

Surprisingly, no study has reported changes in structural volume or connectivity after executive control or attentional training, despite the fact that these types of process-based training have been widely used with older adults. However, an interesting set of studies suggests that structural brain imaging could be used as a biomarker of the impact of attention training. Erickson et al. [17] explored whether the effects of training with the Space Fortress video game could be predicted by the pretraining volume of either of the two key brain regions implicated in learning and memory: the striatum and the hippocampus. They observed that only dorsal striatal volumes, but not ventral striatum, predicted early acquisition rates.

Taken together, these findings suggest that structural volume in certain regions of the brain could help predict effects of cognitive training, and that cognitive training can induce short-term structural changes in older adults. Measures of regional grey matter volume with VBM, cortical thickness and white matter integrity with DTI and FA have been shown to be sensitive to cognitive training in healthy older adults. Though we found no study that had used structural brain imaging as a biomarker of training efficacy in MCI, structural brain changes have been documented in the early course of AD. In particular, regional cortical thickness was found to be reduced in MCI and predicted progression from MCI to AD [18, 19]. This measure might thus represent a very powerful surrogate marker to assess cognitive training effects in MCI populations as well.

## Functional Imaging as a Biomarker of Cognitive Training

Functional brain imaging displays patterns of brain activation as patients perform cognitive tasks. Functional imaging has proven to be a critical tool for understanding the changes in neural mechanisms occurring in aging and AD. For this reason, it has potential as a biomarker. It also has many of the features necessary to qualify as a valid surrogate marker in healthy aging and in early AD. First, functional brain imaging is age-sensitive. When brain imaging is used in older adults to examine changes in cerebral blood flow associated with memory tasks, it reveals a combination of increased activation patterns (eg, prefrontal activation during working memory tasks) and decreased activation patterns (eg, in the left prefrontal and median temporal lobes) or compensatory activation patterns. Compensatory patterns of activation refers to the process by which alternative or atypical brain regions compensate for reduced processing efficiency that occurs with age. Compensatory recruitment can involve the hemispheric homologues (see HAROLD model [hemispheric asymmetry reduction in older adults] proposed by Cabeza [20]) or anterior recruitment (see PASA [posterior–anterior shift in aging] in Davis et al. [21]). Functional brain imaging also shows impairments early in the development of AD [22–24]. Reduced activation is found in the medial temporal areas but also in some regions of the prefrontal cortex of AD patients. Studies of MCI, the early stage of AD, have reported the presence of hyperactivation early in the process, followed by hypoactivation as patients progress from MCI to AD [25–28]. Several studies [25, 29–31] have reported that brain activation differs markedly as a function of the severity of MCI, with greater brain activation found in patients with early phase MCI than at a later stage in the disease. It was also found that hippocampal activation was inversely associated with disease severity [29, 31]. O’Brien et al. [32] showed a marked decrease in activation over a 2-year period during the MCI phase. Thus, whereas AD is mostly characterized by reduced brain activation, a number of studies on MCI have reported increased activation; that is, greater task-related brain recruitment in persons with MCI than in healthy older adults. There is also accumulating evidence that activation is inversely related to the severity of symptoms and disease. Overall, milder symptoms and earlier phases of the disease are characterized by greater task-associated brain recruitment.

Functional brain imaging can thus qualify as a biomarker of aging and AD because it is sensitive to chronological age. It also reveals a characteristic pattern of impairment in those diagnosed with AD that changes with disease severity and progression. A few studies have used functional brain imaging as a biological surrogate of training efficacy in healthy aging populations and persons with MCI. In healthy aging, most studies reporting training-related changes in brain activation patterns have used memory training. Among the first studies to do so, Nyberg et al. [33] noted improved memory performance in both older and younger adults after training with a visual-based mnemonic (the method of loci). They observed increased brain activity (using positron emission tomography [PET]) during memory encoding in occipitoparietal and frontal brain regions after training in younger adults. In older adults, increased occipitoparietal activity was observed only in participants that improved after training, and no change was observed in frontal regions. Valenzuela et al. [34] used localized proton magnetic resonance spectroscopy (MRS) to measure changes in the biochemistry of the right hippocampus, midline parietal–occipital region, and left frontal lobes after memory training with the method of loci. They observed improved performance after 5 weeks of training accompanied by increased creatine and choline signals in the hippocampus. This study shows that focused memory exercises can induce measurable and persisting biochemical changes in the hippocampus, and that use of the memory program may have led to increased resting oxidative phosphorylation in a region that plays a critical role in memory processing.

Training-induced changes in brain activity were also reported after attention control training using computer-based programs. Erickson et al. [35] conducted a randomized longitudinal dual-task training study of functional MRI (fMRI) activation measures in older adults. They observed training-induced changes in activation in two cortical areas commonly associated with age-related atrophy: the dorsal and ventral prefrontal cortex. Interestingly, some brain regions showed equal changes among older and younger adults, whereas others showed differential training effects. For instance, in older adults, increased activity was found in the left ventrolateral prefrontal cortex (VLPFC) region (near Broca’s area), suggesting an increased reliance on verbal or inner speech strategies during dual-task performance. Increased verbalization also has been reported as an efficient strategy to enhance performances in training with a switching task [36]. The authors also observed decreased activity in the right VLPFC in both younger and older adults, suggesting a reduced dependence on response selection strategies or a more efficient response-stimulus association. That both age groups showed similar reductions in activity in this region and that changes in brain activation correlated with changes in behavior suggest that patterns of changes in brain activation were important correlates of training-related improvements in behavior and age is not a factor. Brehmer et al. [37•] examined the behavioral performance and neural activity following 5 weeks of intensive working memory adaptive individualized training. Brain activity was measured before and after training, using fMRI, while participants performed a working memory task in two difficulty conditions. Neocortical brain activity decreased post-training only in the training group, which indicates intervention-related increases in neural efficiency.

A few studies have used functional brain imaging as a biological surrogate of training efficacy in MCI, and those studies indicate that fMRI is sensitive to changes following cognitive training in this group of individuals. Belleville et al. [38••] found that memory-related activation increased after strategy memory training in persons with MCI. Researchers also found that training increased activation in specialized brain regions involved in memory and in compensatory brain regions not typically activated by the verbal memory task (eg, the right inferior parietal lobe). Furthermore, relative to healthy older adults, a number of brain regions that were dysfunctional before training were no longer different following training. Importantly, activation of the right inferior parietal lobe was found to correlate with the efficacy of memory training in persons with MCI. This indicates that the biological change has a valid relation to the expected clinical outcome. Training in this study did not increase activation of the hippocampus, which is surprising considering the importance of this structure in AD. This might be due to the fact that the training program relied heavily on the teaching of visual-based strategies and thus promoted the recruitment of prefrontal and posterior brain regions. Hampstead et al. [39•] used a memory training method that relied on the identification of salient visual cues to learn new face–name associations. In a pilot study, they reported increased activation in the default-mode network, which comprised the medial frontal, parietal, and occipital brain areas, as well as increased connectivity between the temporal lobe, occipital regions, and precuneus following training in MCI populations. In a subsequent study, the researchers used a form of memory training relying on mental imagery to learn object–location associations and compared it to an exposure-control condition [40••]. Before training, they found reduced hippocampal activation in MCI participants relative to controls. Hippocampal activation increased significantly following training compared to the exposure-control condition. Overall the studies of Hampstead et al. [39•, 40••] found changes almost exclusively in areas that showed impaired activation before the training. Rosen and collaborators [41] randomly assigned persons with MCI to either the POSIT science program (Posit Science Corporation, San Francisco, CA), designed to improve speed and temporal auditory processing, or a control condition where patients participated in diverse computerized activities. The authors reported increased left hippocampal activation in trained participants only. There was a tendency for a significant correlation between brain changes and behavioral changes, but this was not significant, perhaps due to the very small sample size.

To our knowledge, only two studies have examined brain-related changes following executive training in MCI participants. Carlson et al. [42] randomly assigned 17 individuals with low education, low income, and low mini–mental state examination to participate in Experience Corps, a program promoting social engagement, or to a control condition. Brain activation associated with performing an executive function test (flanker-task) had increased in the left dorsolateral prefrontal cortex and anterior cingulate gyrus in trained participants. Clare et al. [43] reported a single case study where goal-oriented training of one MCI patient led to decreased activation in sensory regions and increased activation in memory-related regions during an associative face–name association task.

PET can measure neural activity by recording the uptake of [18F]fluorodeoxyglucose (FDG). Reduced FDG-PET at rest may reveal early cerebrometabolic changes in AD and could represent a valid biomarker of neuronal injury in early AD and MCI [44, 45]. A 6-month multicomponent cognitive training program was found to reduce decline in brain glucose metabolism in MCI and early AD participants [46•]. The strongest attenuated decline was found in the left anterior temporal lobe and left anterior cingulate in persons with MCI. However, there was no correlation between cognitive improvement and changes in glucose metabolism. In turn, Small and collaborators [47] found that a multimodal intervention (cognitive stimulation, physical activity, stress reduction, healthy diet) decreased FDG-PET uptake in the left dorsolateral prefrontal cortex of non-demented older adults. This can be interpreted as supporting more efficient processing following training in older adults, whereas the data with MCI might suggest an impact on the pathological process underlying AD.

## Implications for Future Research

The present review of recent studies provides support for the use of structural and functional brain imaging as sensitive surrogate biomarkers for the effects of cognitive training (see Table 1 for summary findings). However, further studies are required to generalize these findings to larger groups and more diverse training protocols. At the structural level, reliable training effects have been reported for regional brain volume, cortical thickness, and white matter microstructure. At the functional level, task-related brain activation (using fMRI and PET) and FDG-PET at rest were found to be sensitive to training effects. In general, cognitive training led to increased brain volume and increased density and coherence of white matter tracts. Functionally, training increased brain metabolism at rest and task-related brain activation in persons with MCI; however healthy older adults showed patterns of increased and decreased activation.

**Table 1:** Summary of findings

Biomarker used	Effect observed
Grey matter volume (VBM)	Increased volume
Cortical thickness	Increased thickness
White matter integrity (DTI)	Increased FA
Biochemistry (MRS)	Increased creatine and choline signal
Glucose metabolism (FDG-PET)	Reduced activation in healthy aging
	Increased activation in MCI
Task-related activation	Increased & decreased activation in healthy aging
	Increased activation in MCI

*VBM* voxel-based morphometry; *DTI* diffusion tensor imaging; *FA* fractional anisotropy; *MRS* magnetic resonance spectroscopy; *FDG-PET* fluorodeoxyglucose positron emission tomography; *MCI* mild cognitive impairment

Apart from being sensitive to change, there are a number of other important characteristics to guide the use of biomarkers as surrogate markers of training efficacy. One of these is its reliability over time. Training efficacy is assessed by repeated measurement, and thus, measures ought to be consistent at the individual and/or group level. Some of the aforementioned studies have implemented a “replication” component in their design to assess changes in an independent group of nontreated participants. For example, the replication component provided by Engvig et al. [14••], though not a reliability study as such, provides support for the use of cortical thickness as a replicable measure in healthy older adults. Studies in young adults have shown that many of the DTI measures have good test–retest reliability when controlling for motion and using a sufficient number of gradient directions [48]. Formal reliability studies have shown that fMRI is reliable for use with older adults and persons with MCI [49, 50]. Clement and Belleville [49] found very few changes in the areas and levels of activation when comparing two measures separated by a 2-month interval, a timeframe typically found in cognitive training studies. Furthermore, it was found that persons with MCI, older adults, and young adults showed comparable overlap ratios, a reproducibility index measuring the amount of voxels activated in one versus both sessions. Importantly, however, the magnitude of the overlap ratio only indicates moderate between-session agreement. This suggests that treatment effects can be more reliably measured by comparing condition contrasts (or region of interest [ROI]) across sessions rather than by comparing voxel activation (see also Putcha et al. [50]). Similarly, intraclass correlations show important intrasubject variability, indicating that while reliable at the group level, care should be taken when interpreting fMRI treatment data at the level of individuals.

One other important characteristic when selecting a “surrogate marker” is that the selected biological measure reflects a clinically relevant change. One way to determine this is by examining the correlation between changes in the biological marker and changes in the relevant clinical or cognitive outcome. In some of these studies, correlations were found between changes in the biomarker and changes in cognitive or clinical status (for example, [15••, 38••, 35]). However, this was not systematically found (for example, [13, 16, 41, 46•]) or even documented. Lack of a relation might be attributable to many factors, which need to be better understood, including insufficient power, inappropriate selection of clinical variables, or the presence of a nonselective effect.

Relevance also can be determined by selecting biomarkers that reflect neuropathological processes underlying the disease, and in that case, it is important to fully understand the relationship between the surrogate marker and the pathophysiology of the condition of interest [10]. Most studies have used markers that are considered valid measures of the processes that underlie normal aging or AD. However, some critical biomarkers of early AD have surprisingly not been tested as surrogate markers of training. One of them is beta-amyloid accumulation. It is unclear whether short-term cognitive training is likely to have an effect on beta-amyloid deposition. However, some recent studies have reported that having engaged in cognitively stimulating activities in early and middle life is associated with reduced beta-amyloid accumulation in older adults [51]. It has been suggested that the increased synaptic activity provided by cognitive stimulation might protect against amyloid deposition.

## Conclusions

In conclusion, this review supports the notion that brain-imaging techniques could be reliably used to assess training-related changes in brain structure and function in healthy older adults and patients with MCI or AD. However, future studies are needed to identify all the potential biomarkers and surrogate biomarkers of brain plasticity induced by cognitive training interventions. We believe that following a structured methodology that meets the prerequisites provided in the present review would effectively guide future research and help to increase the body of knowledge on brain changes associated with cognitive training in older adults.
